# The American and Colonial Journals

**Published:** 1841-01

**Authors:** 


### II. THE AMERICAN AND COLONIAL JOURNALS.

*Coagulation of the Blood fifteen hours after Death.* By R. DUNGLISON, M.D., Philadelphia.

FROM a late number of the Bulletin of the "Proceedings of the American Philosophical Society," (No. 12, for May, June, and July, 18mo, pp. 216,) we extract the following notice:

"Dr. Dunglison gave the particulars of a case in which blood that flowed, on dissection, from the arteries of the brain, coagulated fifteen hours after the death of the individual.

"The patient died after a severe agony, and after an illness of some duration, for which mercury had been administered so as to affect the system freely. On opening the head the arteries of the brain were found turgid with blood, and on removing the brain the blood flowed from them and coagulated.

"Dr. Dunglison made some remarks on the singularity of this phenomenon, and its relations to physiology and medical jurisprudence, and stated that it completely overthrew the views of those who believe that the blood is either possessed of a vital influence or receives some influence from the living vessels that contain it, which maintains its fluidity, and that so soon as it is removed from these influences it coagulates or dies. In this case the blood remained fluid, and coagulation took place fifteen hours after the total cessation of respiration and circulation, and after the blood had become cold; circumstances showing that the phenomena is wholly physical in its nature."

Of this case we had not an opportunity of seeing anything until after death; nor was an accurate history of it attainable. The patient had been delivered about a month previously, and had suffered under symptoms, as it was believed, of peritonitis, for which she was bled generally and locally, and had taken small doses of calomel, which produced severe salivation with considerable ulceration of the gums. Two or three days before her dissolution she was affected with diarrhœa, with heat and dryness of skin, quick and feeble pulse, and considerable stupor, passing her urine and fæces involuntarily. Under these symptoms she gradually sank.

Although the fact that blood may remain fluid in the vessels for a considerable time after death, and coagulate when removed from them, has been noticed before, it does not appear to have given rise to any physiological deductions of moment, yet it is replete with interest to the physiological enquirer.

It is interesting to remark, that in all these cases, we believe, as well as in the one which we observed, mercury had been largely administered, and it may be a topic for farther investigation, whether this peculiarity be in any manner connected with the free use of that or other agents.

*American Med. Library and Intelligencer. August* 1, 1840.

---

[The two following papers, extracted from our most excellent contemporary of Philadelphia, are very valuable, and deserve the notice of surgeons. We wish we possessed similar records from our own great hospitals. We are sorry to be under the necessity of omitting the tabular views of the whole series of cases, having room for the results only.]

I. *Statistical Account of the Cases of Amputation performed at the Pennsylvania Hospital from January* 1, 1838, *to January* 1, 1840. By G. W. Norris, M.D., one of the Surgeons to the Institution.

All those amputations in which the operation was performed within twenty-four hours after admission, are included under the head of immediate, the patient in such cases having been brought to the house soon after the receipt of his injury. With one exception, the common circular operation was performed, and the stumps were all dressed so as to procure union by the first intention. The ordinary mode of dressing is first to bring the flaps together by means of three or four long strips of adhesive plaster, and after covering the lips of the wound with lint spread with cerate, to apply a small cushion of charpie over the extremity of the stump, and to secure the whole with a bandage moderately tight. The first dressing was generally made on the third or fourth day, and repeated daily afterwards till cicatrization was complete.

Of eighty amputations on seventy-nine patients, performed during a term of ten years at the Pennsylvania Hospital, thirty-five were primary, of which twenty-four were cured and eleven died, four of the deaths occurring within the twenty-four hours immediately following it.

Twenty were secondary, of which thirteen were cured and seven died.

Twenty-five were for the cure of chronic affections, of which twenty were cured and four died.

Thirty-two of the amputations were of the upper extremity, of which twenty-seven were cured and five died.

Forty-seven were of the lower extremity, of which thirty-one were cured and sixteen died.

Seven were amputations at the joints, of which four were cured and three died.

| | | | |
|---|---|---|---|
| 13 of the 79 operated on, | were under 20 years of age, of whom | 12 were cured and | 1 died. |
| 26 | between 20 and 30, | 19 | 7 |
| 22 | between 30 and 40, | 15 | 7 |
| 16 | between 40 and 50, | 9 | 7 |
| 2 | upwards of 50, | 2 were cured. | |
| — | | — | — |
| 79 | | 57 | 22 |

The conclusions to be drawn from an analysis of the two tables which I have now published are,

1. That amputation with us is to be regarded as an operation attended with much danger to the life of the individual, the mortality after it being 1 in $3\frac{7}{11}$.

2. That the chances of success after it are much greater in persons who have been for some time suffering from chronic diseases, than in those who have it done while enjoying robust health, the mortality in the former class of cases being 1 in $6\frac{1}{4}$, while in the latter it is 1 in $3\frac{2}{11}$.

3. That immediate amputations after injuries are less fatal than secondary operations, the mortality after the former being 1 in $3\frac{2}{11}$, while in the latter it is 1 in $2\frac{6}{7}$.

4. That amputation of the lower extremity is much more fatal than that of the superior member, the mortality after the former being 1 in $2\frac{15}{16}$, while in the last-mentioned class of cases it is only 1 in $6\frac{2}{5}$.

5. That the danger increases with the age of the individual operated on.

### II. *Statistics of the Amputations of Large Limbs that have been performed at the Massachusetts General Hospital; with Remarks.* By GEO. HAYWARD, M.D., one of the Surgeons to the Hospital.

IN a large proportion of the following cases, the amputation was done by the circular incision; the flap operation was adopted occasionally, whenever there was reason to believe that a better stump could be made by it than by the other method. The dressings were always of a light and simple kind, consisting of two or three strips of adhesive plaster and a small compress and roller; and yet there are some surgeons of the present day, who would perhaps regard these as more cumbersome than was necessary. If the bleeding was slight, the dressings were applied before the patient left the operating room; but if there was anything more than an oozing from the veins, it was deferred till a few hours after. Secondary hemorrhage was not frequent, though it sometimes occurred; pressure was generally sufficient to arrest it, but occasionally it was found necessary to open the stump, and tie one or more vessels. In one case where hemorrhage occurred twelve days after the operation, from a diseased state of the posterior tibial artery, the femoral artery was tied. No one who had secondary hemorrhage died, and though it sometimes debilitated the patient, in no case was there any permanently injurious effect from it. In all the cases it was attempted to heal the wound by the first intention, and in a few instances it was completely successful, but in by far the greater number it was only partially so. It has not been the usual practice at the Massachusetts Hospital to administer an opiate before an operation, though in a few instances it has been done. In one case where amputation was performed on a patient with delirium tremens, twelve grains of opium were given shortly before the operation; he became drowsy soon after and recovered.

From this table, it appears that there were seventy operations on sixty-seven patients, three patients having two limbs removed. In one of these three cases, one operation was above and the other below the knee, and in the other two, both operations were below; the first patient died, and the other two did well.

Of the whole number operated on, fifteen died and the remainder recovered, at least so far as to be able to leave the hospital, though it is probable that in some instances the disease may have returned.

There were thirty-four patients who had the thigh amputated, and one of these had the other leg taken off at the same time below the knee; of this number, nine died. Of twenty-three patients whose legs were amputated below the knee, two having both legs removed, five died; and of the ten who had an arm amputated, six below and four above the elbow, one died.

This goes to confirm the prevailing opinion among surgeons, that amputation of the lower extremities is more often followed by fatal consequences than that of the upper, and that death takes place more frequently after amputation of the thigh, than after that of the leg. More than a quarter of those whose thighs were amputated died, while there was but little more than one death in five among those whose legs were removed below the knee, and only one of the ten whose arms were amputated. This patient too died of delirium tremens. The operation to be sure did not arrest the disease, but apparently contributed nothing to the fatal result.

This table tends also to support the opinion, that patients who undergo amputation for chronic diseases are much more likely to recover than those in whom it is performed in consequence of recent accidents. Of the first class, there were forty-five patients afflicted with various diseases, and of this number all recovered but six; and of the remaining twenty-two, whose limbs were removed on account of recent injuries, no less than ten died, being nearly half of the latter and less than one in seven in the former.

This fact certainly gives support to the opinion, that a state of high health is not favorable to surgical operations; and it also tends to show that death after amputation is not by any means attributable in all cases to the operation alone; for if it were, the proportion of deaths should be as large among one class of patients as among the other. There can be no doubt, I think, that the result is influenced very much, not only by the age and constitution of the patient, and the disease or injury for which the operation is performed, but also by the period at which it is done. I have before said that I thought that amputation was "often performed when it might have been avoided." But this remark applies principally to cases of recent injury. In those of chronic diseases of the limbs, the error is more apt to be of the opposite character; the operation is either not performed, or if done at all, frequently not till it is too late. It cannot be denied, I think, that there is a disposition at the present day to defer amputation too long in cases of diseased limbs; there is an unwillingness to admit that the morbid affection is beyond the reach of remedies, and the operation is too often postponed till other parts become affected, or the system is worn down by continued irritation. At length the limb is removed; but the patient, already exhausted by disease and long suffering, is hurried to his end by the very means that might have saved him, if they had been earlier employed.

If amputation is frequently too long delayed in chronic diseases of the limbs, it is, I fear, very often resorted to in recent injuries earlier than it should be. Many limbs that have been removed, might probably have been saved; but where this cannot be done, it is rare that much inconvenience would follow from a little delay.

In most cases of accident sufficiently severe to justify amputation, the whole system has suffered a great shock, and an operation at this time, before reaction is fairly established, is very likely to cut off what little chance the patient might otherwise have of recovery. While the extremities are cold and the action of the heart is feeble, the local injury is hardly, if at all, perceived, and adds nothing to the patient's sufferings. An operation cannot be required then; and yet how often it is done at that period, the better judgment of the surgical attendant sometimes being overruled by the importunate interference of the bystanders.

If the injury be not so serious as to cause almost immediate death, reaction usually comes on with proper management in a few hours, and then, if an operation be necessary, it can be done with a much greater prospect of success.

With regard to the ages of the patients operated on, it appears that there were

| | | | | | | | |
|---|---|---|---|---|---|---|---|
| | | Under 20 | years of age | 13, | of this number | 1 | died. |
| Over 20 | and not exceeding | 30 | ,, | 31, | ,, | 8 | ,, |
| ,, 30 | ,, | 40 | ,, | 9, | ,, | 3 | ,, |
| ,, 40 | ,, | 50 | ,, | 10, | ,, | 2 | ,, |
| ,, 50 | ,, | 60 | ,, | 3, | ,, | 1 | ,, |
| | | Over 70 | ,, | 1, | ,, | 0 | ,, |

Whole number, 67, No. of deaths, 15.

*American Journal of Medical Sciences. May,* 1840.

*On the Effects of Temperance Societies in diminishing Sickness and Mortality among the Troops in India.* By WM. BELL, Surgeon of the Cameronians, Calcutta.

THE Temperance Society of the Cameronians was established about two years ago, and though the numbers have fluctuated considerably, yet upon the whole it has been well supported, and there can be no doubt that its influence has been most favorable both on the health and on the morals of the regiment. In December, 1837, the average monthly strength had been 121; and in December, 1838, it remained 159, having fluctuated in the mean time from 208 to 108. These alterations are to be accounted for, in some degree, by the arrival of detachments of invalids and recruits, which generally interrupt for a time the steadiness of the corps The effect in health during 1837, was that of the society the per centage of sick amounted to 3 $\frac{1}{5}$, and of the rest of the regiment to 101$\frac{3}{4}$. During 1838, the average daily sick of the society has been 6$\frac{1}{2}$ per cent., and of the remainder of the regiment 9 per cent.

These results, however gratifying, do not convey an adequate idea of the benefits of the society; for a number of men whose constitutions had been ruined by dissipation became members, and several such remained in hospital nearly the whole year, until they were invalided. The admissions of the last year have been of the society 1 in 25, and of the remainder of the regiment 1 in 11.

This is the first instance I believe of any regiment stationed in Fort William, having established a regular Temperance Society, and it therefore becomes a duty to point out such favorable circumstances in the state of the corps, as may fairly be attributed, more or less, to its influence, in order that others may be induced to seek for the same results by the adoption of similar institutions.

1st. The deaths in the regimental hospital have been in 1837, 26, and in 1838, 22, whereas the average mortality in Fort William, for a period of fourteen years previously, had been seventy-two nearly.

2d. The spirits drunk in the canteen have been for 1837, 9673 gallons less, and for 1838, 8242 gallons less than the regiment was entitled to draw.

3d. During the above two years, the beer sold in the canteen amounts to 156 hogsheads, 46$\frac{1}{2}$ gallons, and the wine to 326 dozen.

This consumption of beer and wine is not quoted as very creditable or praiseworthy, but as a proof that the use of spirits, in a great degree, was abandoned, even by those who did not choose to be particularly abstemious with regard to their beverage.

4th. The remittances by the men to their friends at home, and to the Edinburgh Savings' Bank, were for 1837, £587 18*s.* 9*d.*, and for 1838, £763 4*s.* 6*d.*

This is not quoted as a remarkably large sum, being smaller than those of previous years, but as a proof that even on half batta stations, and where the temptations to spend money are numerous, the soldier who can avoid dissipation has both the means and the inclination to provide, to a certain degree, for the future comfort of himself or his friends.

5th. Since the arrival of the regiment in India, it appears that the consumption of spirits has diminished from the enormous quantity of 10,000, 12,000, and 14,000 gallons to 2516.

What is the cause of this remarkable change?

It may depend on more causes than one; but there can be no doubt that the chief is the establishment of a Temperance Society, and its principles.

6th. There are other circumstances which deserve to be mentioned, such as the decrease of liver complaints from 111, 140, and 135, as in the years 1832-33-34, to 82, and to 50, as during the last year; but this may be partly owing to other causes, besides the disuse of ardent spirits, as to change of climate, from the upper to the lower provinces, &c.; yet a comparison of the tables will show the great superiority in regard to health in the Cameronian over any other corps that has ever been stationed in Fort William.

7th. As Temperance Societies have been formed in most of the Queen's corps serving in Bengal, the following abstract is here added, showing the result of the whole from the 1st January to 30th June, 1838, as drawn up by the Inspector General.

ABSTRACT OF THE COMPARATIVE STATE OF HEALTH OF THE TEMPERANCE SOCIETIES OF HER MAJESTY'S TROOPS SERVING IN BENGAL, FROM JANUARY 1 TO JUNE 30, 1838.

| Months. | Strength of the Temperance Society. | Strength of the remainder of the Regiment. | Relative proportions admitted to Strength. | | Average number of men daily in Hospital of the Society. | Per centage of the Society. | Average number of men daily in Hospital not of the Society. | Per centage not of the Society. |
|---|---|---|---|---|---|---|---|---|
| | | | Temperance Society. | Remainder of Regiment. | | | | |
| Jan. | 1953 | 2569 | 1 in 18·77-104 | 1in 9·220-261 | 49·24-31 | 2, 54 | 206·27-31 | 8, 15 |
| Feb. | 1840 | 2639 | 1 in 20·10-110 | 1 in 9·245-266 | 41·20-28 | 2, 27 | 218·11-28 | 8, 27 |
| March | 1542 | 2879 | 1 in 14·44-107 | 1 in 7·149-390 | 45·12-31 | 2, 94 | 248·18-34 | 8, 66 |
| April | 1359 | 3081 | 1 in 10·9-135 | 1 in 5·261-564 | 74·12-30 | 5, 47 | 316·22-30 | 10, 28 |
| May | 1282 | 3161 | 1 in 18·44-69 | 1 in 6·353-468 | 67·6-31 | 5, 24 | 336·30-31 | 10, 66 |
| June | 1364 | 3065 | 1 in 19·53-69 | 1 in 6·371-349 | 62·3-30 | 4, 55 | 317·6-30 | 10, 35 |
| Total | 9340 | 17354 | 1 in 16·47-143 | 1 in 7·284-1199 | 341·40-84 | 3, 65 | 1771·731-930 | 10, 20 |

[The preceding paper comprises only a small part of the evidence communicated to the public on this vital subject, and all having the same bearing. If *total abstinence* from intoxicating drinks generally should be substituted in India for the abstinence from spirituous liquors only, we have no doubt but the results would be greatly more striking still.]

*India Journal of Medical Science*. *Nov.* 1839.

